# 12-Month prevalence of hypertension in Germany

**DOI:** 10.17886/RKI-GBE-2017-016

**Published:** 2017-03-15

**Authors:** Hannelore Neuhauser, Ronny Kuhnert, Sabine Born

**Affiliations:** Robert Koch Institute, Department for Epidemiology and Health Monitoring, Berlin, Germany

**Keywords:** HYPERTENSION, ADULTS, GERMANY, HEALTH MONITORING, GEDA

## Abstract

Hypertension is among the most important risk factors for cardiovascular diseases and therefore a significant determinant of the most frequent causes of death in adults. According to the GEDA 2014/2015-EHIS survey nearly one in three adults in Germany have self-reported physician-diagnosed hypertension. Men are affected more in the age group of under 65 year olds. Nearly two thirds of all men and women aged 65 and over have hypertension. An educational gradient is particularly evident among women, with a higher prevalence of self-reported hypertension among women with low levels of education. Compared to the German average, prevalence of self-reported hypertension among men is higher in Mecklenburg-Western Pomerania and Saxony-Anhalt and among women in all East German federal states with the exception of Berlin. Only in Bremen is the prevalence among men lower than the national average.

## Introduction

Hypertension is among the most important risk factors for cardiovascular diseases and therefore a significant determinant of the most frequent causes of death in adults. Hypertension is mostly due to a combination of genetic predispositions, age, gender and various unhealthy diet and living conditions such as excess weight, high salt intake, high alcohol consumption, lack of exercise and stress. Only rarely is hypertension the result of other diseases. Hypertension is, however, a risk factor that patients can significantly influence through lifestyle changes and consistent drug therapy [1].

## Indicator

For the GEDA 2014/2015-EHIS survey, participants were asked to answer three standardised questions on hypertension either online or in writing. The survey defined people who responded that their doctor had diagnosed them at least once with hypertension and who confirmed that they had either suffered from hypertension during the past twelve months or were currently taking medicines to reduce blood pressure as people with self-reported physician-diagnosed hypertension during the past twelve months. Blood pressure values were not surveyed.

The analysis is based on the answers given by 23,967 participants aged eighteen and over (49 participants were excluded on grounds that they failed to fill in all the required fields). Answers were adjusted to account for differences between sample structure and the overall German population (on 31 December 2014) with regard to gender, age, type of community and levels of education. Education levels were defined using a standardised procedure (International Standard Classification of Education, ISCED) that takes into account educational and professional qualifications [2]. A detailed description of the methodology used by GEDA 2014/2015-EHIS can be found in the article ‘German Health Update – new data for Germany and Europe’ [3] in this issue.

**GEDA 2014/2015-EHIS**

**Table:** 

**Data holder:**	Robert Koch Institute
**Aims:**	to provide reliable information about the population’s health status, health-related behaviour and health care in Germany, with the possibility of a European comparison
**Method:**	questionnaires completed on paper or online
**Population:**	people aged 18 years and above with permanent residency in Germany
**Sampling:**	registry office sample; randomly selected individuals from 301 communities in Germany were invited to participate
**Participants:**	24,016 people (10,872 men; 13,144 women)
**Response rate:**	26.9%
**Study period:**	November 2014 – July 2015
**Data protection:**	all participants were informed about the study’s aims and content and about data protection, and provided their informed consent

More information is available at www.geda-studie.de

## Results and discussion

According to the GEDA 2014/2015-EHIS survey results, nearly one in three adults (30.9% of women and 32.8% of men) have self-reported, physician-diagnosed hypertension (table 1). Prevalence of self-reported hypertension increases with age. Nearly two thirds of those aged 65 and over (63.8% of women and 65.1% of men) have self-reported hypertension.

An association between self-reported hypertension and education exists for women of all age groups. Women in the high education group are significantly less likely to report physician-diagnosed hypertension than women from the low education group. For men, a similar association exists for those aged between 45 and 64.

**Fig. 1:**
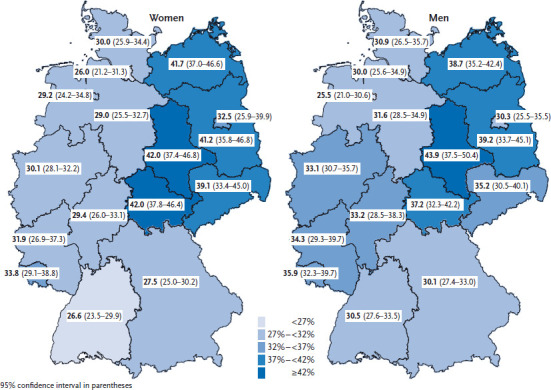
12-month prevalence of self-reported, physician-diagnosed hypertension among women and men according to German federal state Source: GEDA 2014/2015-EHIS

Split by regions, prevalence of self-reported hypertension is higher among men in Mecklenburg-Western Pomerania and Saxony-Anhalt and among women in all East German federal states with the exception of Berlin compared with the national average. Prevalence among men is lower than the German average in Bremen (figure 1).

Population-wide examination surveys to collect standardised blood pressure measurements are expensive and require considerable efforts, which is why they are conducted only at larger intervals. Interview surveys such as GEDA 2014/2015-EHIS, however, can be conducted at shorter intervals and show the prevalence of self-reported hypertension. Yet the fact of being self-reported depends decisively on three factors: people’s awareness of hypertension (which can only be determined through examination surveys); prevalence of hypertension in the population (including undetected hypertension); as well as the methodological particularities of such a survey (in particular the operationalisation of the term hypertension and the information provided on the drug treatment of hypertension, as patients with controlled hypertension are likely to answer that they do not suffer from hypertension).

According to the results of the last national examination survey, the German Health Interview and Examination Study for Adults (DEGS1, 2008–2011), over 80% of participants with hypertension were aware of the condition. Awareness was greater among women than men (86.8% and 78.3% respectively) and higher among older than among younger adults [4]. Prevalence in GEDA 2014/2015-EHIS of self-reported hypertension is higher than the prevalence of known hypertension in DEGS1 2008-2011 [4]. DEGS1 prevalences, however, refer only to adults up to 79 years. In addition, the DEGS1 definition of known hypertension required not only self-reported physician-diagnosed hypertension but also hypertensive blood pressure values or the intake of anti-hypertensive medication. Moreover, DEGS1 measures point prevalence, whereas prevalence in GEDA 2014/2015-EHIS is the twelve-month prevalence. An increase in the prevalence of hypertension is unlikely, as data from numerous surveys reveals a continuous and consistent decrease in blood pressure levels in Germany and Western Europe in general over the past two decades [1, 4-6].

Compared to GEDA 2012 results [7], the twelve-month prevalence of self-reported physiciandiagnosed hypertension is slightly higher in GEDA 2014/2015-EHIS. This is potentially due to methodological differences between the two surveys. Whereas both studies defined the indicator similarly as regards taking into account the medicines patients were given and how the indicator was limited to only cases of physician-diagnosed hypertension, the wording and order of questions in the two studies were different so that the surveys are not fully comparable.

The indicator for self-reported hypertension analysed here consists of three questions on hypertension. It expands and more precisely defines the simple self-reporting of hypertension during the past twelve months, as collected by the European Health Interview Survey (EHIS). This ensured that 621 participants (2.5% of those surveyed), who answered that they had not suffered from hypertension during the past twelve months, were nonetheless assigned to the correct category, as they also stated that they had in the past been diagnosed with hypertension by a physician and were currently taking medicines to control their blood pressure. These appear to be cases of controlled hypertension where patients no longer consider themselves as having hypertension. A more specific question seems desirable that asks whether hypertension was (ever) diagnosed by a physician because interviews do not define the term hypertension and participants’ answers are therefore subject to multiple influences that cannot be further determined.

The greater prevalence of self-reported hypertension observed in some East German federal states matches results from the DEGS1 survey [8]. It should be noted that the positive developments in north-eastern Germany, which have led to greater awareness, more frequent treatment and better control of hypertension [1], cannot be demonstrated with the indicator self-reported hypertension in GEDA 2014/2015-EHIS (since higher awareness increases the prevalence of self-reported hypertension while better treatment and control help reduce the risk in the population, however, have no influence on the prevalence of self-reported hypertension).

Higher prevalence of self-reported hypertension among women with lower levels of education is in accordance with GEDA-2012 results [7], as well as with DEGS1 findings of an association between the prevalence of hypertension and socioeconomic status [9]. Analyses based on DEGS1 data, however, reveal that people’s socioeconomic status has no influence on hypertension awareness, treatment or control.

Overall, there is a lack of nationwide population-based data on blood pressure in Germany [1]. In the intervals between examination surveys, monitoring self-reported hypertension through interview surveys such as GEDA 2014/2015-EHIS can detect current trends in self-reported hypertension. However, because of the limited validity of self-reported hypertension in interview surveys, it will require standardised blood pressure measurements and an operational and reproducible definition of hypertension to confirm these trends [10]. Increasing validity by including information on medicines and limiting the category to physician-diagnosed hypertension should be discussed when further developing EHIS indicators that currently use only self-reported hypertension in the past twelve months as an indicator for hypertension.

A comprehensive comparison of the blood pressure situation in Germany and between European countries, however, requires an analysis of different blood pressure indicators, including not only data from interviews but also standardised measurements of mean systolic and diastolic blood pressure to determine the prevalence of known and undetected hypertension as well as data on the degree of awareness, treatment and control of hypertension. A simple cross-country comparison of self-reported hypertension during the past twelve months (the current EHIS indicator for hypertension, as described in the article ‘Health monitoring and health indicators in Europe’ [11] in this issue, which does not take into account whether a person is taking medicines to lower blood pressure and that does not limit the sample to people with physician-diagnosed hypertension) reveals great differences between countries, possibly mainly grounded in methodology. It was therefore proposed to expand and better define the current EHIS indicator. Current international and systematic reviews based on examination surveys [6, 12] show a comparatively high prevalence of hypertension in Germany despite the high rate of controlled hypertension and the decrease in mean blood pressure in the population. These international analyses show that monitoring blood pressure at the population level is complex. In Germany, a consortium has conducted a comprehensive analysis of blood pressure with data of the Robert Koch Institute’s federal health monitoring as well as data from population-based regional surveys. These analyses should be continued [1].

## Key statements

- According to the GEDA 2014/2015-EHIS survey nearly one in three adults in Germany have self-reported physician-diagnosed hypertension.
- The prevalence of self-reported hypertension increases with age. Nearly two thirds of people aged 65 and over have been diagnosed with hypertension.
- Women with higher levels of education are significantly less likely to report physician-diagnosed hypertension than women with lower levels of education. A similar association exists for men aged between 45 and 64 years.
- Compared to the German average, prevalence of self-reported hypertension is higher among men from Mecklenburg-Western Pomerania and Saxony-Anhalt, and higher among women in all East German federal states with the exception of Berlin.

## Figures and Tables

**Table 1:** 12-month prevalence of self-reported, physician-diagnosed hypertension according to gender, age and educational status (n=23,967) Source: GEDA 2014/2015-EHIS

**Women**	**%**	**(95%-CI)**	**Men**	%	**(95%-CI)**
**Women total**	**30.9**	**(29.8-32.1)**	**Men total**	**32.8**	**(31.6-33.9)**
**18 – 29 Years** Low education Medium education High education	4.2 6.5 4.0 1.4	(3.1-5.6) (3.9-10.5) (2.7-5.8) (0.7-3.0)	**18 – 29 Years** Low education Medium education High education	4.4 5.4 4.5 2.3	(3.3-6.0) (3.2-8.9) (3.1-6.6) (1.0-5.2)
**30 – 44 Years** Medium education High education Low education	9.0 12.3 9.6 5.6	(7.8-10.4) (8.2-18.0) (8.0-11.4) (4.3-7.3)	**30 – 44 Years** Low education Medium education High education	14.5 12.7 17.5 9.9	(12.8-16.5) (8.5-18.5) (14.9-20.3) (7.9-12.2)
**45 – 64 Years** Low education Medium education High education	31.6 37.4 32.0 25.4	(29.9-33.5) (33.1-41.8) (29.7-34.3) (22.8-28.2)	**45 – 64 Years** Low education Medium education High education	38.3 42.7 40.1 33.5	(36.4-40.1) (37.9-47.7) (37.4-42.8) (30.9-36.2)
**≥ 65 Years** Low education Medium education High education	63.8 66.4 62.9 58.0	(61.5-66.1) (62.8-69.9) (59.4-66.2) (53.4-62.4)	**≥ 65 Years** Low education Medium education High education	65.1 65.5 65.2 64.5	(62.9-67.1) (60.5-70.3) (61.9-68.4) (61.4-67.5)
**Total (women and men)**	**31.8**	**(31.0-32.7)**	**Total (women and men)**	**31.8**	**(31.0-32.7)**

CI=Confidence interval

## References

[1] Neuhauser H Diederichs C Boeing H. (2016) Hypertension in Germany. Data from seven population-based epidemiological studies (1994–2012). Dtsch Arztebl Int 113(48):809-815 28073425 10.3238/arztebl.2016.0809 PMC5241792

[2] Eurostat (2016) International Standard Classification of Education (ISCED) http://ec.europa.eu/eurostat/statistics-explained/index.php/Glossary:International_standard_classification_of_education_%28ISCED%29/ (As at 01.03.2017)

[3] Saß AC Finger JD Allen J. (2017) German Health Update: New data for Germany and Europe. The background to and methodology applied in GEDA 2014/2015-EHIS. Journal of Health Monitoring 2(1):75-82 www.rki.de/journalhealthmonitoring 10.17886/RKI-GBE-2017-023 PMC10161278 37151302

[4] Neuhauser HK Adler C Rosario AS. (2015) Hypertension prevalence, awareness, treatment and control in Germany 1998 and 2008-11. J Hum Hypertens 29(4):247-253 25273858 10.1038/jhh.2014.82

[5] Finger JD Busch MA Du Y. (2016) Time Trends in Cardiometabolic Risk Factors in Adults. Dtsch Arztebl Int 113(42):712-719 27866566 10.3238/arztebl.2016.0712 PMC5143790

[6] Collaboration NCDRF (2017) Worldwide trends in blood pressure from 1975 to 2015: a pooled analysis of 1479 population-based measurement studies with 19.1 million participants. Lancet 389(10064):37-55 27863813 10.1016/S0140-6736(16)31919-5 PMC5220163

[7] Robert Koch-Institut (RKI) (2014) Ergebnisse der Studie “Gesundheit in Deutschland aktuell 2012”. Beiträge zur Gesundheitsberichterstattung des Bundes. RKI, Berlin http://www.rki.de/DE/Content/Gesundheitsmonitoring/Gesundheitsberichterstattung/GBEDownloadsB/GEDA12.pdf?__blob=-publicationFile (As at 01.03.2017)

[8] Diederichs C Neuhauser H (2014) Regional variations in hypertension prevalence and management in Germany: results from the German Health Interview and Examination Survey (DEGS1). J Hypertens 32(7):1405-1413 24834980 10.1097/HJH.0000000000000211

[9] Neuhauser H Sarganas G (2015) High blood pressure: a concern for everyone. Publ. Robert Koch Institute, Berlin GBE kompakt 6(4) https://www.rki.de/EN/Content/Health_Monitoring/Health_Reporting/GBEDownloadsK/2015_4_high_blood_pressure.pdf;j-sessionid=7998E3A7184F63A497D2B2B9C9215282.2_cid290?__blob=publicationFile (As at 01.03.2017)

[10] Tormo MJ Navarro C Chirlaque MD. (2000) Validation of self diagnosis of high blood pressure in a sample of the Spanish EPIC cohort: overall agreement and predictive values. EPIC Group of Spain. J Epidemiol Community Health 54(3):221-226 10746117 10.1136/jech.54.3.221 PMC1731632

[11] Fehr A Lange C Fuchs J. (2017) Health monitoring and health indicators in Europe. Journal of Health Monitoring 2(1):3-21 www.rki.de/journalhealthmonitoring 10.17886/RKI-GBE-2017-020.2 PMC10161272 37151308

[12] Mills KT Bundy JD Kelly TN. (2016) Global Disparities of Hypertension Prevalence and Control: A Systematic Analysis of Population-Based Studies From 90 Countries. Circulation 134(6):441-450 27502908 10.1161/CIRCULATIONAHA.115.018912 PMC4979614

